# Introducing the Dementia Crisis Service in East Kent: Can We Reduce Rates of Hospital Admission?

**DOI:** 10.1192/bjo.2024.516

**Published:** 2024-08-01

**Authors:** Swarupa SriBalaskanda, Bastiaan Veugelers

**Affiliations:** Kent and Medway NHS and Social Care Partnership Trust, Maidstone, United Kingdom

## Abstract

**Aims:**

Admissions to hospital can be traumatic for a person with dementia due to an inability to cope with unfamiliar environments, faces and routines. The dementia crisis service provides a rapid response and support, in particular to carers and care providers. The team support in managing problematic behaviours to avoid the need for a hospital admission. The team can complete physical examination and bloods in the home environment, reducing the need for involvement of further clinicians.

This project aims to evaluate the effectiveness of the dementia crisis service in reducing admissions to mental health wards.

**Methods:**

The pilot for the service began in January 2023. We looked at the number of admissions to Heather ward, an older adult mental health ward in Canterbury, East Kent (the base of the team) over a 5 month period, between August 2023 – January 2024. We compared this to admission numbers a year ago. We looked at what proportion of patients were admitted with behavioural and psychological symptoms of dementia (BPSD), over the same time period to evaluate whether the number of BPSD admissions has changed since the existence of the team.

**Results:**

The number of overall admissions to Heather ward decreased from 43 to 32. The number of detained patients remained the same, 13 patients over the 5 month period. Looking more closely at the nature of some of the hospital admissions, a referral was not made to the crisis service for some of the admitted patients.

**Conclusion:**

The team have been providing this service for just over a year, including the three month pilot. The limited data does not show enough evidence that the crisis service reduces rates of hospital admission. As this is a new service, there is much work to be done to increase the profile of the team. We would like to re-evaluate the admission data after more information has been disseminated to referrers about the service and the support they offer.

